# Ceramide analog C2-cer induces a loss in insulin sensitivity in muscle cells through the salvage/recycling pathway

**DOI:** 10.1016/j.jbc.2023.104815

**Published:** 2023-05-11

**Authors:** Cécile L. Bandet, Sophie Tan-Chen, Sarah Ali-Berrada, Mélanie Campana, Maxime Poirier, Agnieszka Blachnio-Zabielska, Jean-Paul Pais-de-Barros, Claude Rouch, Pascal Ferré, Fabienne Foufelle, Hervé Le Stunff, Eric Hajduch

**Affiliations:** 1 Centre de Recherche des Cordeliers, INSERM, Sorbonne Université, Paris, France; 2 Institute of Cardiometabolism and Nutrition, ICAN, Assistance Publique-Hôpitaux de Paris, Paris, France; 3 CNRS UMR 9197, Institut des Neurosciences Paris-Saclay, CNRS UMR 9197, Université Paris-Saclay, Saclay, France; 4 Epidemiology and Metabolic Disorders Department, Medical University of Bialystok, Bialystok, Poland; 5 Lipidomics Core Facility, INSERM UMR1231 - Université Bourgogne Franche Comté, Dijon, France; 6 Functional and Adaptive Biology Unit, UMR 8251, CNRS, Université de Paris Cité, Paris, France

**Keywords:** Akt PKB, cell signaling, diacylglycerol, lipid signaling, lipogenesis, signal transduction, metabolism, lipotoxicity, oleate

## Abstract

Ceramides have been shown to play a major role in the onset of skeletal muscle insulin resistance and therefore in the prevalence of type 2 diabetes. However, many of the studies involved in the discovery of deleterious ceramide actions used a nonphysiological, cell-permeable, short-chain ceramide analog, the C2-ceramide (C2-cer). In the present study, we determined how C2-cer promotes insulin resistance in muscle cells. We demonstrate that C2-cer enters the salvage/recycling pathway and becomes deacylated, yielding sphingosine, re-acylation of which depends on the availability of long chain fatty acids provided by the lipogenesis pathway in muscle cells. Importantly, we show these salvaged ceramides are actually responsible for the inhibition of insulin signaling induced by C2-cer. Interestingly, we also show that the exogenous and endogenous monounsaturated fatty acid oleate prevents C2-cer to be recycled into endogenous ceramide species in a diacylglycerol O-acyltransferase 1–dependent mechanism, which forces free fatty acid metabolism towards triacylglyceride production. Altogether, the study highlights for the first time that C2-cer induces a loss in insulin sensitivity through the salvage/recycling pathway in muscle cells. This study also validates C2-cer as a convenient tool to decipher mechanisms by which long-chain ceramides mediate insulin resistance in muscle cells and suggests that in addition to the *de novo* ceramide synthesis, recycling of ceramide could contribute to muscle insulin resistance observed in obesity and type 2 diabetes.

Insulin resistance plays a major role in the pathogenesis of type 2 diabetes (T2D). Skeletal muscles are quantitatively the largest glucose users in response to insulin and are considered as the main effectors for the development of insulin resistance (1). It is now clear that circulating free fatty acids (FAs), highly increased in T2D, are key players for the development of muscle insulin resistance, and high plasma concentrations of FAs are generally associated with an increased risk of developing diabetes (2). In healthy individuals, free FAs are stored as lipid droplets in adipocytes. In situations like obesity and T2D, FAs coming from both lipolysis and food intake are in excess and eventually accumulate in insulin tissues (liver, skeletal muscles).

Ectopic fat accumulation is associated with insulin resistance, a phenomenon called lipotoxicity. However, FA themselves are not directly involved but rather their metabolic derivatives such as ceramides or diacylglycerols (DAGs) (3, 4). In the context of obesity-associated FA overload, ceramides are mainly produced *de novo* from saturated FAs (SFAs) and particularly palmitate. This synthesis occurs in the endoplasmic reticulum (ER) and begins with the condensation of *L-*serine with palmitoyl-CoA by serine palmitoyltransferase (SPT), generating 3-keto-dihydrosphingosine, reduced to dihydrosphingosine by 3-keto-dihydrosphingosine reductase. Dihydrosphingosine is then acylated by ceramide synthase (CerS) isoforms to form dihydroceramide species (3). The 4-5 position of the sphingoid base backbone is finally desaturated by dihydroceramide desaturase-1 to form different ceramide species (5). Interestingly, ceramide can also be synthetized through a salvage pathway which involves the acylation of sphingosine by CerS isoforms (6). Sphingosine is only generated from the degradation of complex sphingolipids such as glycosphingolipids or deacetylation of ceramide in lysosomes by ceramidases. Then, sphingosine can be re-acylated by CerS into ceramide (6). Thus, through this salvage pathway, sphingolipids can be recycled to give new molecules of ceramide. Interestingly, this pathway was shown to contribute from 50 to 90% of sphingolipid biosynthesis (6), and a growing body of evidence is also starting to point toward roles of this pathway in many biological responses (6, 7).

Many studies including ours showed that ceramides play a crucial role in lipotoxicity-induced insulin resistance (3) and demonstrated that downregulating *de novo* ceramide biosynthesis prevents their deleterious action on insulin signaling in peripheral tissues (3, 8, 9). In contrast, only few studies showed that ceramides supplied from the salvage pathway are implied as potential actors of lipotoxicity in peripheral tissues (10, 11, 12).

Ceramide species are involved in many processes such as regulation of apoptosis, cell differentiation, and insulin signaling (3). In the liver, C16-ceramide species are harmful for insulin sensitivity (13). Conversely, very-long chain ceramide species (C22/C24/C24:1) appear to exert protective functions in hepatocytes (14, 15). In muscle cells, most of ceramide species are increased in response to lipotoxicity (3, 4, 16, 17), but the species that mediates insulin resistance is still not clear, even if C18-ceramide is often associated to insulin resistance in this tissue (9, 18, 19, 20). Mechanism by which ceramides act negatively on the insulin signaling pathway has been well characterized in muscle cells (4). To summarize, ceramides rapidly target and inhibit Akt through the activation of either the phosphatase PP2A or the PKC ζ (16, 21, 22, 23), and in longer term, they target and inhibit insulin-induced insulin receptor substrate-1 activity in a PKR/JNK– and/or Prep1-p160–dependent manner (24, 25).

Most of the studies investigating ceramide effects used biologically active, cell-permeable, short-chain ceramide analogs such as C2- or C6-ceramide species (17, 22, 25, 26, 27, 28, 29). These ceramide species are easy to use *in vitro*, but their structure differs from more “physiological” counterparts (30). Even if the presence of C2-ceramide (C2-cer) was established in mouse brain and liver, its concentration is approximately 5000-fold less than long-chain ceramide species (31) usually used to induced lipotoxic responses (17, 32, 33, 34).

Since C18-ceramide is the predominant ceramide species in skeletal muscle and since skeletal muscle CerS1-derived C18-ceramide promotes insulin resistance (19), how can C2-cer reproduce the deleterious action of endogenous C18-ceramides in muscle cells?

The goal of the present study was to evaluate whether C2-cer needs to be converted into longer chain ceramide species in muscle cells using the sphingolipid salvage pathway in order to induce insulin resistance.

## Results

### Endogenous long chain ceramide species are generated in response to exogenous C2-cer

**Figure 1:**
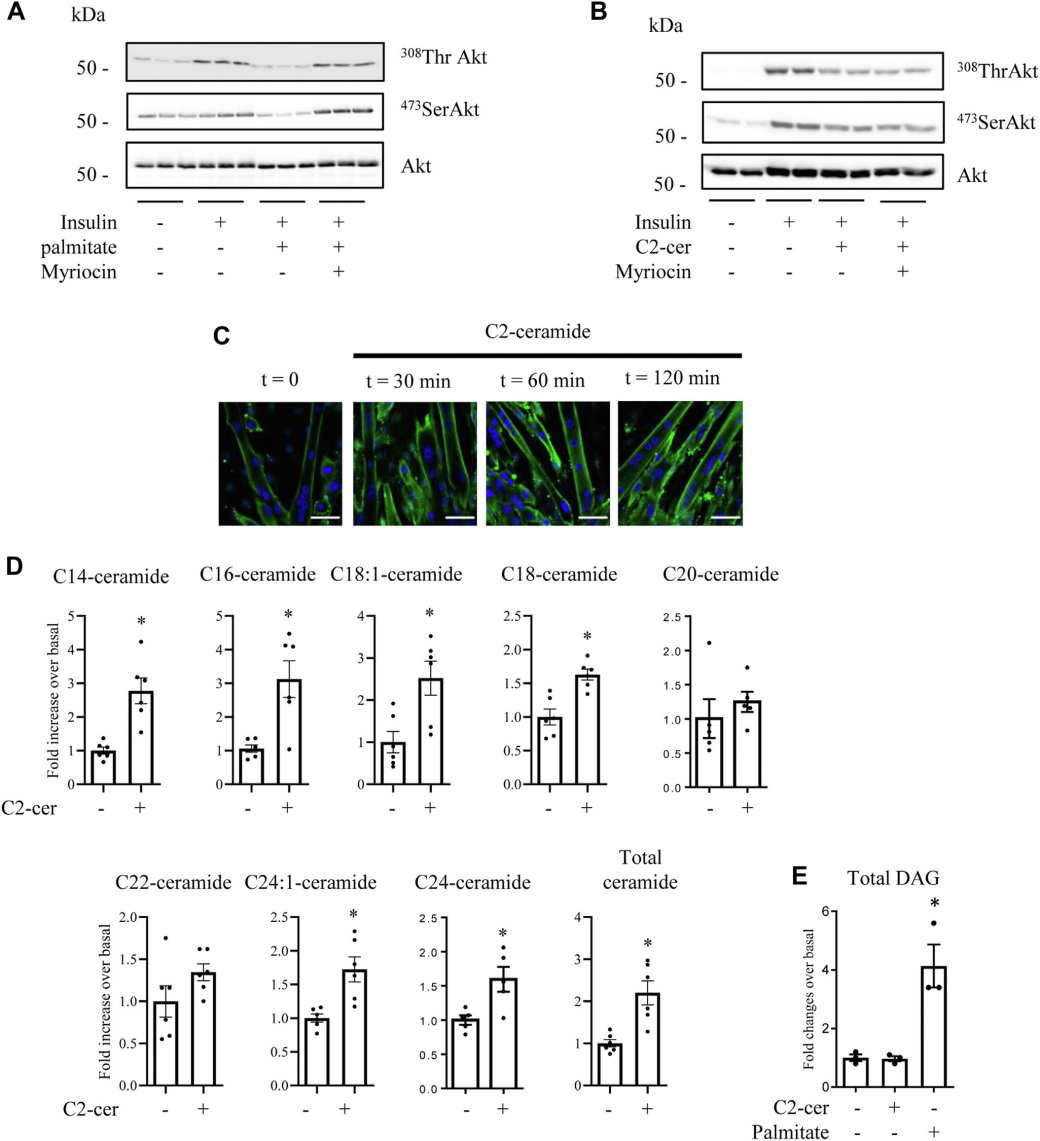
**Effect of C2-cer on endogenous ceramide species synthesis in C2C12 myotubes.***A*, C2C12 myotubes were incubated for 16 h with 0.75 mmol/l palmitate in the presence or not in the presence of 10 μmol/l myriocin followed by 100 nmol/l insulin 10 min before cell lysis. *B*, C2C12 myotubes were incubated with 100 μmol/l C2-cer for 2 h followed by 100 nmol/l insulin 10 min before cell lysis. Cell lysates were immunoblotted with the indicated antibodies. *C*, C2C12 myotubes were incubated with 50 μmol/l C2-cer from 30 min to 2 h and stained for ceramide using an anti-ceramide antibody (*green*). In parallel, nuclei were stained with DAPI (*blue*). Scale bar represents 50 μm. *D*, C2C12 myotubes were incubated with 100 μmol/l C2-cer for 2 h. Following these incubations, muscle cells were harvested to assess ceramide content as described in the Experimental procedures section. Results are mean ± SEM (n = 5–6). ∗ represents significant change *p* ≤ 0.05 relative to the untreated control myotubes. *E*, C2C12 myotubes were incubated with 100 μmol/l C2-cer for 2 h or with 0.75 mmol/l palmitate for 16 h. Then, DAG content was assessed. Results are mean ± SEM (n = 3). ∗ represents significant change *p* ≤ 0.05 relative to the untreated control. C2-cer, C2-ceramide; DAG, diacylglycerol.

When incubated with palmitate, C2C12 myotubes become rapidly insulin resistant, as shown by the inhibition of insulin-induced phosphorylation of Akt (Fig. 1*A*). Preincubation of cells with myriocin, a potent and specific inhibitor of SPT, the rate limiting enzyme of *de novo* ceramide synthesis (17, 32), completely prevented the inhibitory action of palmitate, demonstrating that palmitate inhibits insulin signaling through *de novo* production of ceramide. Short-chain C2-cer mimicked the effect of palmitate on C2C12 myotubes. Indeed, C2-cer incubation strongly reduced insulin-induced Akt phosphorylation (Fig. 1*B*). However, pretreatment of cells with myriocin did not prevent C2-cer–induced inhibition of Akt phosphorylation by insulin (Fig. 1*B*).

In order to analyze whether C2-cer was used as a backbone to generate endogenous ceramide species, we incubated C2C12 myotubes with C2-cer up to 2 h and endogenous ceramide species were quantified. First, we detected ceramide intracellular content by immunofluorescence using an anticeramide antibody that specifically detects both C16- and C24-ceramide species (35). Figure 1*C* shows that, in the basal state, very low content of both ceramide species was detected in muscle cells. Addition of C2-cer induced a rapid and robust increase in the concentration of both C16- and C24-ceramide species in C2C12 myotubes (Fig. 1*C*). Then, we refined these results by assessing intracellular ceramide species concentrations using ultra-high-performance liquid chromatography tandem mass spectrometry (UHPLC/MS/MS). Figure 1*D* shows that incubation of C2-cer for 2 h of C2C12 myotubes increased several endogenous long chain ceramide species such as C14:0, C16:0, C18:1, C18:0, C24:0, and C24:1-ceramide. Overall, C2-cer increased the level of endogenous ceramides by around 2-fold (Fig. 1*D*). This is in line with what was observed previously in C2C12 myotubes treated 16 h with palmitate (9).

In parallel, we tested in our cell model the action of another short-chain ceramide species (C6-ceramide, C6-cer), first ceramide species shown to be remodeled into longer-chain endogenous ceramides in cells (36). First, Fig. S1*A* shows that C6-cer inhibited the insulin response in a dose-dependent manner. Furthermore, and as observed with C2-cer (Fig. 1*D*), C6-cer was remodeled into several longer-chain ceramide species (Fig. S1*B*). This result shows that this intracellular recycling mechanism is not limited to one ceramide analog.

Interestingly, and in contrast with palmitate, no increase in DAG content was observed in response to C2-cer treatment (Fig. 1*E*), confirming that the deleterious action of exogenous C2-cer on insulin signaling remains a ceramide-driven process in muscle cells.

### C2 ceramide is deacylated and re-acylated into endogenous ceramide species in C2C12 myotubes

Generation of endogenous long-chain ceramide species from C2-cer could happen through a C2-cer deacylation/re-acylation process that has already been shown to occur on short-chain ceramide in a human lung adenocarcinoma cell line (36).

**Figure 2:**
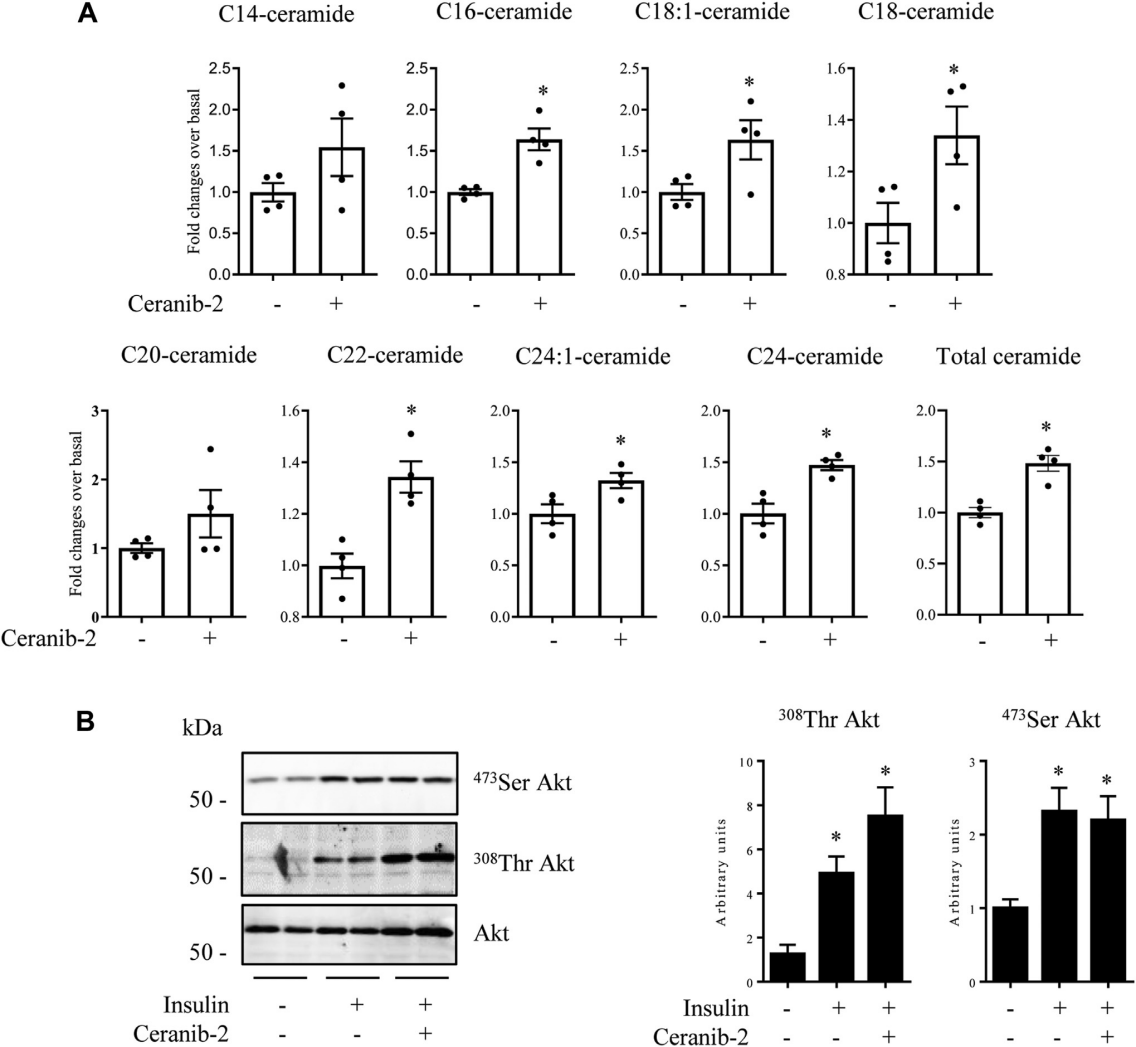
**Effect of ceranib-2 on endogenous ceramide synthesis and insulin response.***A*, C2C12 myotubes were incubated with 50 μmol/l ceranib-2 for 2 h, and ceramide species content was assessed. Results are mean ± SEM (n = 4). ∗ represents significant change *p* ≤ 0.05 relative to the untreated control. *B*, C2C12 myotubes were incubated with 50 μmol/l ceranib-2 for 2 h and with or without 100 nmol/l insulin for 10 min before being lysed. Cell lysates were immunoblotted with the indicated antibodies. Scanning densitometry was performed to quantify changes in ^473^Ser Akt and ^308^Thr Akt abundance in cell lysates. Bars represent mean ± SEM (n = 3–4). ∗ represents significant change *p* ≤ 0.05 relative to the untreated control myotubes.

We first tested whether the expression of enzymes involved in this mechanism could be modulated in response to C2-cer. Fig. S2 shows that neither the expression of the six CerS isoforms nor that of the three ceramidase isoforms are significantly modified after 2 h treatment with C2-cer. Then, we treated C2C12 myotubes with ceranib-2, a nonlipid ceramidase inhibitor (37), and after 2 h treatment, we assessed ceramide content in cells. Ceranib-2 alone induced a 50% accumulation in basal total endogenous ceramide concentrations (Fig. 2*A*). It is interesting to note that, like C2-cer, ceranib-2 increased most ceramide species (except C14:0 and C20:0, Fig. 2*A*). Unexpectedly, ceranib-2–induced ceramide content within cells did not cause any default in insulin response, as insulin-induced Akt phosphorylation remained identical with or without the ceramidase inhibitor (Fig. 2*B*).

**Figure 3:**
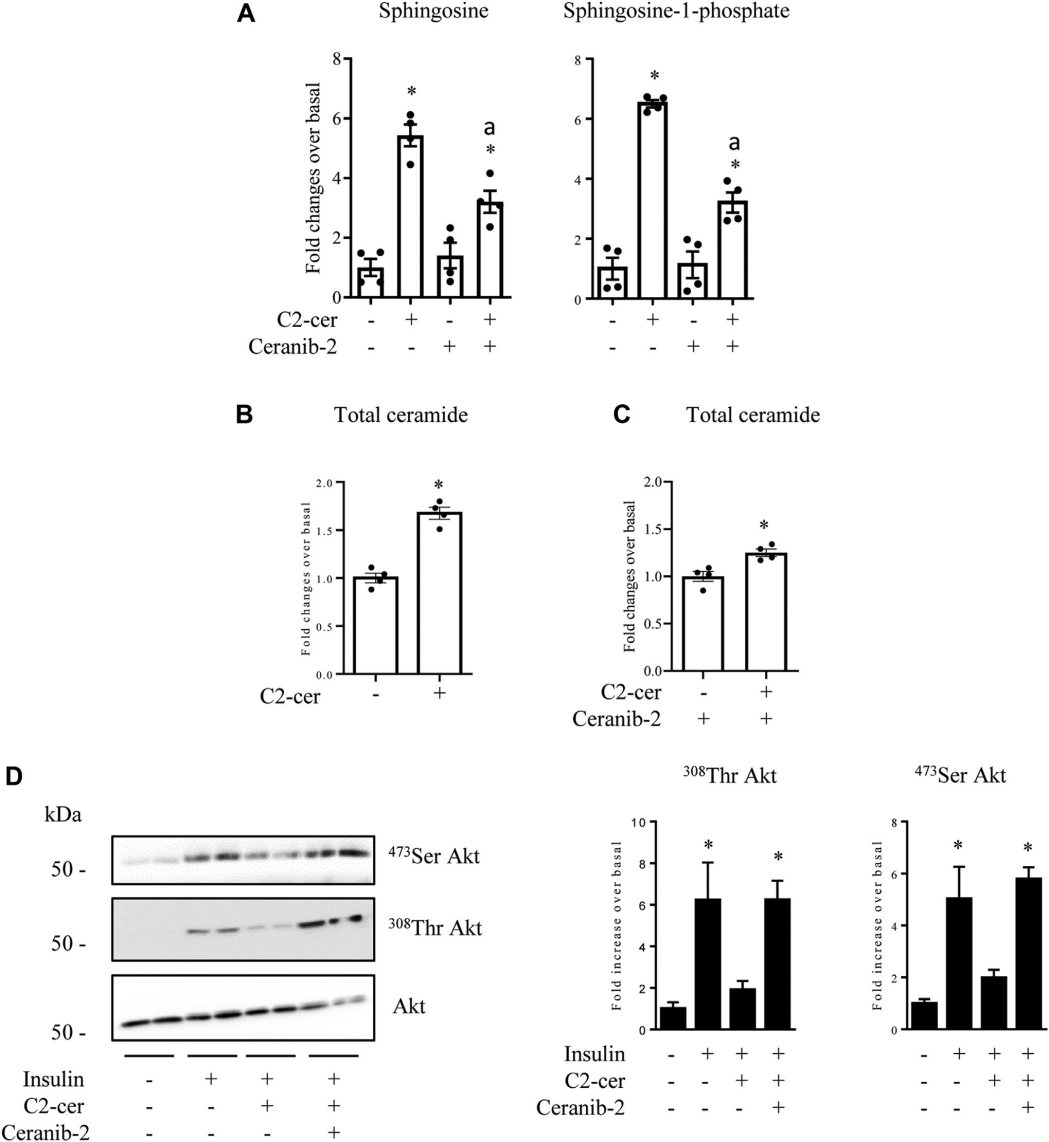
**Effect of ceranib-2 on C2-cer–induced endogenous ceramide synthesis and insulin response.***A*, C2C12 myotubes were incubated with 100 μmol/l C2-cer for 2 h in the presence or not in the presence of 50 μmol/l ceranib-2 (added 30 min before). Sphingosine content was analyzed. Results are mean ± SEM (n = 4). ∗ represents significant change *p* ≤ 0.05 relative to the untreated control. ^a^ represents significant change *p*≤ 0.05 relative to the C2-cer–treated cells. *B*, C2C12 myotubes were incubated with 100 μmol/l C2-cer for 2 h. Total ceramide content was assessed. Results are mean ± SEM (n = 4). ∗ represents significant change *p* ≤ 0.05 relative to the untreated control. *C*, C2C12 myotubes were incubated with 100 μmol/l C2-cer for 2 h in the presence of 50 μmol/l ceranib-2, and total ceramide levels were assessed. Results are mean ± SEM (n = 4). ∗ represents significant change *p* ≤ 0.05 relative to the untreated control. *D*, C2C12 myotubes were incubated with 100 μmol/l C2-cer for 2 h in the presence of 50 μmol/l ceranib-2, followed by 100 nmol/l insulin 10 min before cell lysis. Cell lysates were immunoblotted with the indicated antibodies. Scanning densitometry was performed to quantify changes in ^473^Ser Akt and ^308^Thr Akt abundance in cell lysates. Bars represent mean ± SEM (n = 3). ∗ represents significant change *p* ≤ 0.05 relative to the untreated control myotubes. C2-cer, C2-ceramide.

Then, we checked whether preventing C2-cer deacylation into sphingosine would stop C2-cer–induced endogenous ceramide synthesis and thus its inhibitory action on insulin signaling. Figure 3*A* shows that both sphingosine and sphingosine-1-phosphate (S1P) concentrations were increased following C2-cer addition, demonstrating that C2-cer was converted into both sphingolipids in muscle cells. Ceranib-2 significantly reduced C2-cer–induced sphingosine and S1P content (Fig. 3*A*). In addition, C2-cer treatment alone induced a 60% increase in total ceramide content in muscle cells (Fig. 3*B*), but in this case, and in opposite to what was observed in response to ceranib-2 alone (Fig. 2), a sharp inhibition of the insulin signal was observed in the presence of C2-cer (Fig. 3*D*). Interestingly, inhibition of ceramidase activity with ceranib-2 reduced C2-cer–induced endogenous ceramide content (residual 25% increase, Fig. 3*C*), confirming that endogenous ceramide species synthetized from C2-cer occurred to a large extent through a salvage ceramide pathway in C2C12 myotubes. In this situation, the ceranib-2 inhibitor restores the insulin response in a dose-dependent manner (Figs. 3*D* and S3*A*), suggesting that newly ceramide species synthetized from C2-cer were responsible for the loss of insulin signal.

**Figure 4:**
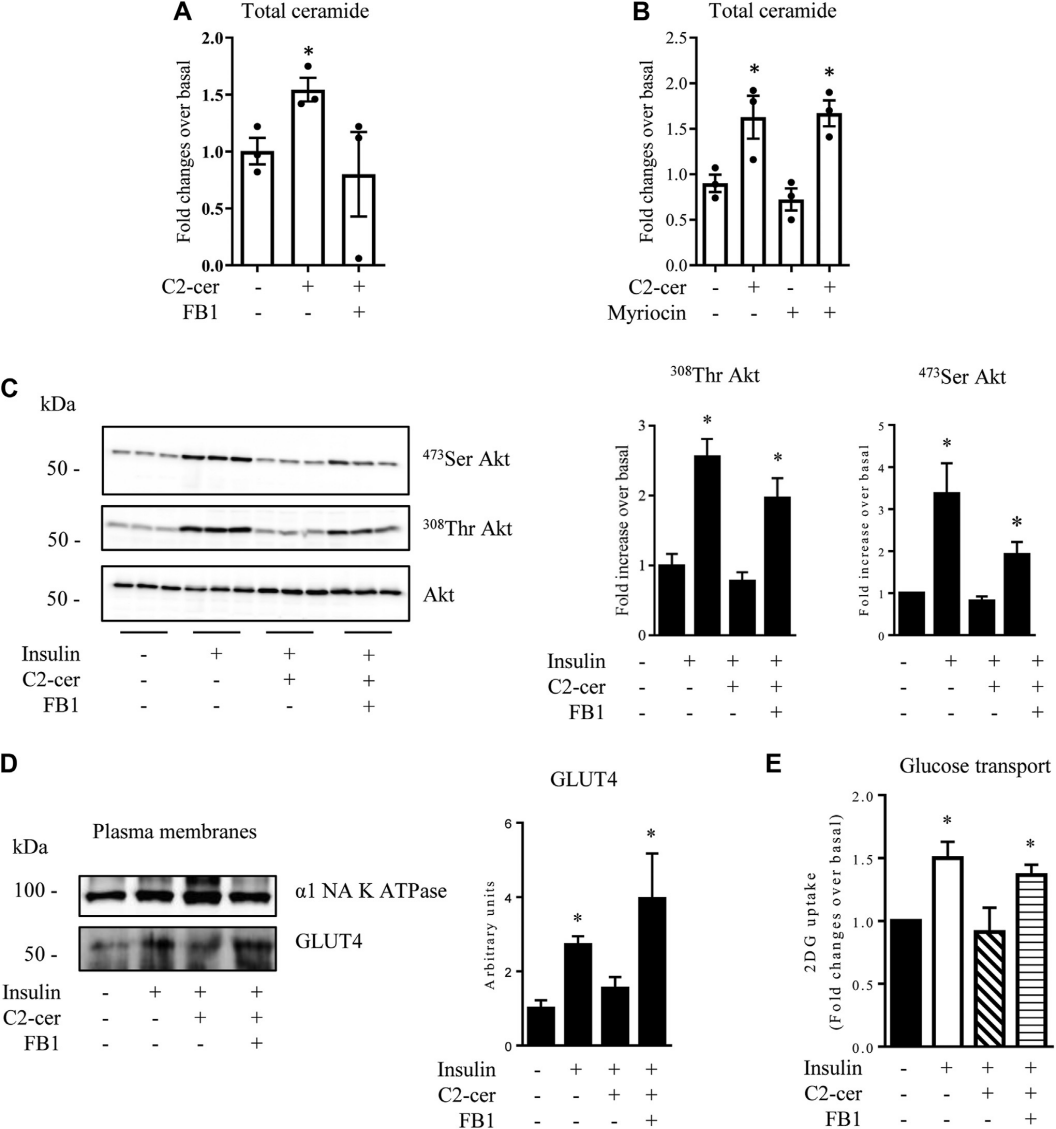
**Effect of C2-cer and FB1 on the insulin signaling pathway in C2C12 myotubes.** C2C12 myotubes were incubated with 100 μmol/l C2-cer for 2 h in the presence or not in the presence of 1 μmol/l FB1 (*A*) or of 10 μmol/l myriocin (*B*) (both FB1 or myriocin added 30 min before), and total ceramide content was analyzed. Results are mean ± SEM (n = 3). ∗ represents significant change *p* ≤ 0.05 relative to the untreated control myotubes. *C*, C2C12 myotubes were incubated with 100 μmol/l C2-cer for 2 h in the presence or not in the presence of 1 μmol/l FB1, followed by 100 nmol/l insulin for 10 min before cell lysis. Scanning densitometry was performed to quantify changes in ^473^Ser Akt and ^308^Thr Akt abundance in cell lysates. Results are mean ± SEM (n = 3). ∗ represents significant change *p* ≤ 0.05 relative to the untreated control myotubes. *D*, C2C12 myotubes were incubated with 100 μmol/l C2-cer for 2 h in the presence or not in the presence of 1 μmol/l FB1, followed by 100 nmol/l insulin for 20 min before cell harvesting and subcellular fractionation as described in the Experimental procedures section. Isolated plasma membranes were immunoblotted with the indicated antibodies. Scanning densitometry was performed to quantify changes in GLUT4 abundance in plasma membranes. Results are mean ± SEM (n = 3). ∗ represents significant change *p* ≤ 0.05 relative to the untreated control myotubes. *E*, C2C12 myotubes were incubated with 100 μmol/l C2-cer for 2 h in the presence or not in the presence of 1 μmol/l FB1, followed by 100 nmol/l insulin for 20 min. At the end of the incubation period, 2-DG uptake was assessed as described in the Experimental procedures section. Results are mean ± SEM (n = 4). ∗ represents significant change *p* ≤ 0.05 relative to the untreated control myotubes. C2-cer, C2-ceramide; FB1, fumonisin B1.

To confirm a sphingosine re-acylation process in the insulin resistance mediated by C2-cer, we treated muscle cells with fumonisin B1 (FB1), an inhibitor of CerS, enzymes that would add different chain length fatty acyl-CoAs to the short chain ceramide-derived sphingosine backbone to generate newly synthetized ceramide species (36). Figure 4*A* shows that FB1 prevented the generation of total endogenous ceramide in response to C2-cer, suggesting that sphingosine derived from C2-cer is undeniably used as backbone to produce other ceramide species in cells. In contrast, and as a negative control, inhibition of SPT with myriocin did not affect C2-cer–induced endogenous ceramide synthesis (Fig. 4*B*). We checked whether FB1 would prevent C2-cer–inhibited insulin signaling in C2C12 myotubes. We treated C2C12 myotubes with C2-cer in the presence or not in the presence of FB1 for 2 h and then with insulin for the last 10 min of the incubation process, and we assessed Akt phosphorylation status. We observed the usual loss in the insulin response with C2-cer (Fig. 4*C*). Interestingly, pretreatment of cells with FB1 prevented the negative action of C2-cer and restored the ability of insulin to induce Akt phosphorylation in a dose-dependent manner (Figs. 4*C* and S3*A*). Altogether, these data confirm that long chain ceramide species generated from C2-cer through a deacylation/re-acylation process are responsible for the loss in insulin signal observed after C2-cer treatment.

Then, we evaluated whether preventing C2-cer–induced generation of long chain ceramide species would have consequences on glucose metabolism downstream of insulin signaling. Figure 4*D* shows that, as previously described in muscle cells (38), insulin induces the translocation of the insulin-regulated glucose transporter (GLUT4) to the plasma membrane where it facilitates glucose uptake into the cell. However, in the presence of C2-cer, GLUT4 could not be recruited anymore to the plasma membrane (Fig. 4*D*). As expected, inhibition of endogenous long chain ceramide generation from C2-cer by FB1 prevented completely the inhibitory effect of C2-cer on insulin-induced GLUT4 translocation to the plasma membrane (Fig. 4*D*). Finally, we assessed the effect of inhibiting CerS activity on insulin-induced glucose uptake. As shown in Figure 4*E*, inhibition of insulin-induced 2-deoxy-glucose (2-DG) uptake by C2-cer was prevented when CerS activity was inhibited by FB1.

### Endogenous ceramide coming from exogenous C2-cer are synthetized from a lipogenic source

**Figure 5:**
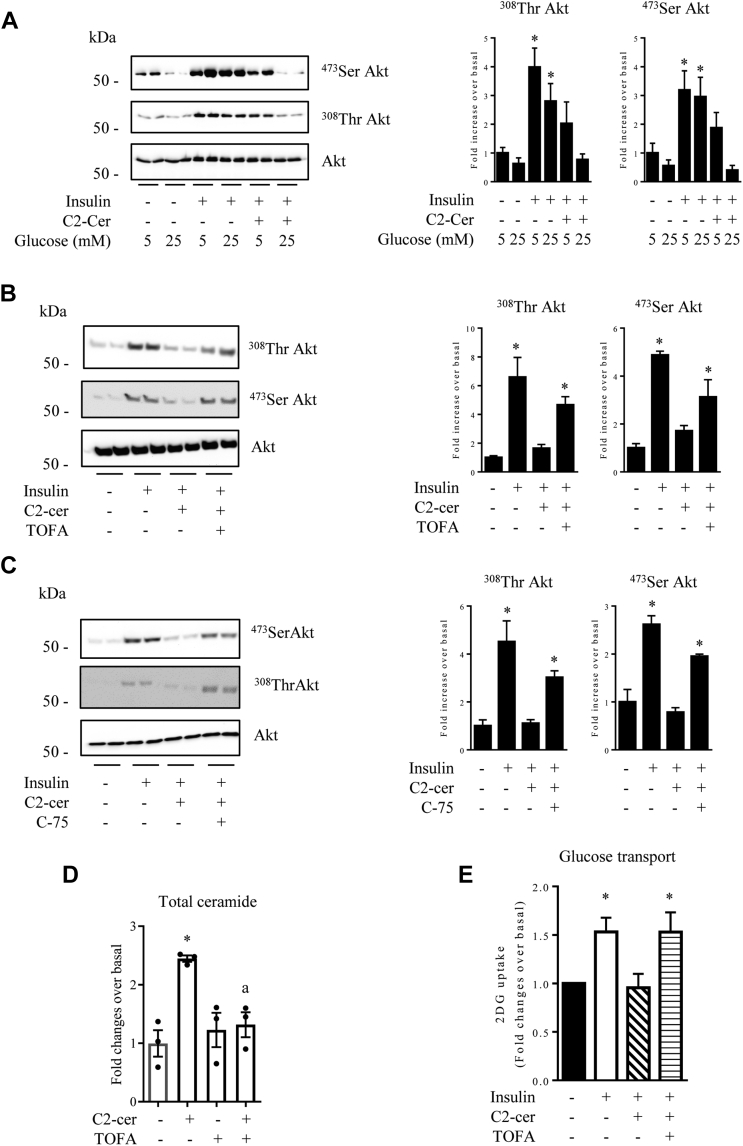
**Inhibition of lipogenesis prevents the deleterious action of C2-cer on insulin signaling in C2C12 myotubes.***A*, C2C12 were cultured in either 5 mmol/l or 25 mmol/l glucose-containing medium for 16 h before addition of 100 μmol/l C2-cer for 2 h and 100 nmol/l insulin for the last 10 min. Then, cells were harvested and lysates were immunoblotted with the indicated antibodies. Scanning densitometry was performed to quantify changes in ^473^Ser Akt and ^308^Thr Akt abundance in cell lysates. Bars represent mean ± SEM (n = 3). ∗ represents significant change *p* ≤ 0.05 relative to the untreated control myotubes. C2C12 myotubes were incubated with 100 μmol/l C2-cer for 2 h with or without 4 μg/ml TOFA (*B*) or 25 μmol/l C-75 (*C*) (both added 30 min before) and with 100 nmol/l insulin for the last 10 min. Then, cells were harvested and lysates were immunoblotted with the indicated antibodies. Scanning densitometry was performed to quantify changes in ^473^Ser Akt and ^308^Thr Akt abundance in cell lysates. Bars represent mean ± SEM (n = 3). ∗ represents significant change *p* ≤ 0.05 relative to the untreated control myotubes. *D*, C2C12 myotubes were incubated with 100 μmol/l C2-cer for 2 h with or without 4 μg/ml TOFA, and total ceramide content was analyzed. Results are mean ± SEM (n = 3). ∗ represents significant change *p* ≤ 0.05 relative to the untreated control myotubes. ^a^ represents significant change *p* ≤ 0.05 relative to the C2-cer–treated myotubes in the absence of TOFA. *E*, C2C12 myotubes were treated with 100 μmol/l C2-cer for 2 h in the presence or not in the presence of 4 μg/ml TOFA, followed by 100 nmol/l insulin for 20 min. At the end of the incubation period, 2-DG uptake was assessed as described in the Experimental procedures section. Results are mean ± SEM (n = 6). ∗ represents significant change *p* ≤ 0.05 relative to the untreated control myotubes. C2-cer, C2-ceramide; TOFA, 5-tetradecyloxy-2-furoic acid.

T2D is a prevalent metabolic disorder characterized by hyperglycemia (glucotoxicity) and hyperlipidemia (lipotoxicity). High circulating glucose concentrations were shown to potentiate the effect of lipotoxicity through ceramide synthesis (8). Hyperglycaemia is also responsible for FA synthesis through the lipogenic pathway in several tissues, including muscle (39). Thus, the FA-synthetized *de novo* could serve as precursors for endogenous ceramide formation from sphingosine. To test this hypothesis, we incubated muscle cells with 25 mM or 5 mM glucose 16 h before to add C2-cer and to check the insulin response. At 25 mM glucose, C2-cer prevented completely insulin-induced Akt phosphorylation (Figure 5*A*). However, at 5 mM glucose, C2-cer action was unable to alter the insulin signal (Fig. 5*A*). Then, we inhibited lipogenesis by using an inhibitor of acetyl-CoA carboxylase, 5-tetradecyloxy-2-furoic acid (TOFA) (40). Acetyl-CoA carboxylase is a key enzyme in lipogenesis that catalyzes the irreversible carboxylation of acetyl-CoA coming from glucose metabolism to generate malonyl-CoA. We followed the insulin response in C2C12 cells that were treated with C2-cer in the presence or absence of TOFA. TOFA completely prevented C2-cer inhibition of the insulin response in a dose-dependent manner (Figs. 5*B* and S3*B*). To confirm these data, we repeated the same protocol by using an FA synthase (FAS) inhibitor, C-75 (41). FAS is another crucial enzyme that catalyzes the *de novo* biosynthesis of long-chain FAs using acetyl-CoA and malonyl-CoA. As already observed with TOFA, inhibition of lipogenesis through the blockade of FAS activity with the C-75 inhibitor totally prevented the inhibition of insulin signaling by C2-cer in a dose-dependent manner (Figs 5*C* and S3*B*).

Then, we assessed total ceramide content in C2C12 cells that were treated with C2-cer in the presence of absence of TOFA. Figure 5*D* shows that TOFA prevented the increase in total ceramide content usually observed in response to C2-cer. Finally, we assessed 2-DG uptake in these cells, and Figure 5*E* shows that the inhibitory action of C2-cer on insulin-induced glucose uptake was reversed in the presence of TOFA.

### Oleate downregulates C2-cer–induced endogenous ceramide species synthesis in muscle cells

Several studies showed that monounsaturated FAs, and in particular oleate, possess the property of preventing endogenous ceramide biosynthesis in muscle cells, mainly by pushing FA such as palmitate towards β-oxidation and triglyceride (TAG) production (42, 43, 44). Oleate was also shown to downregulate the activity of some enzymes of the *de novo* ceramide biosynthesis pathway such as SPT, KDSR, CerS and desaturase-1 (11, 45), suggesting that oleate may reduce the ceramide generation by directly inhibiting its *de novo* synthesis. Our next question was as follows: since C2-cer recycling needs available free FAs to give endogenous ceramide species, could oleate negatively modulate this action?

**Figure 6:**
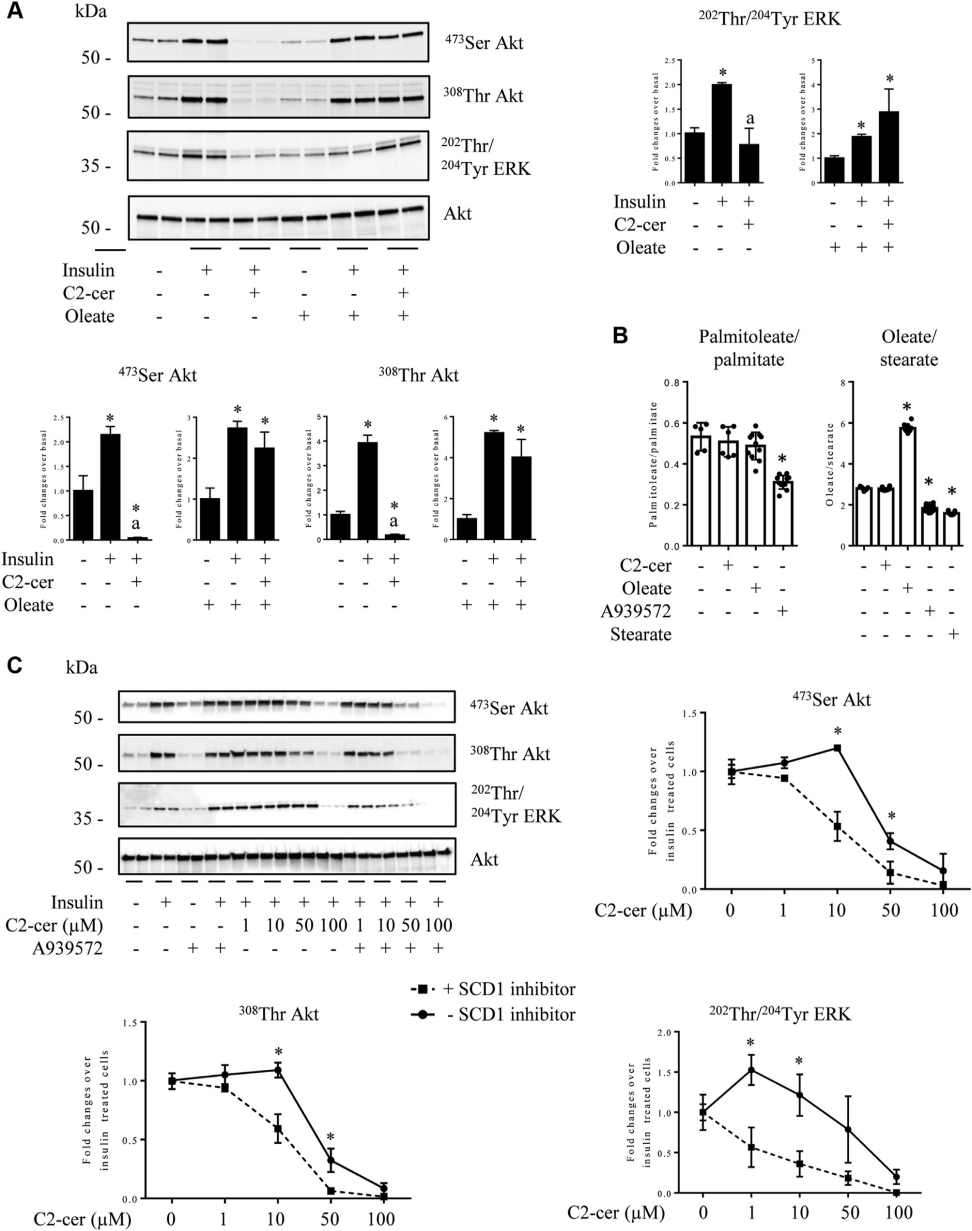
**Oleate prevents the deleterious action of C2-cer on insulin signaling in C2C12 myotubes.***A*, C2C12 myotubes were incubated with 100 μmol/l C2-cer for 2 h with or without 0.3 mM oleate (added 30 min before) and with 100 nmol/l insulin the last 10 min. Then, cells were harvested and lysates were immunoblotted with the indicated antibodies. Scanning densitometry was performed to quantify changes in ^473^Ser Akt, ^308^Thr Akt, and ^202^Thr/^204^Tyr ERK abundance in cell lysates. Bars represent mean ± SEM (n = 3). ∗ represents significant change *p* ≤ 0.05 relative to the untreated control myotubes. ^a^ represents significant change *p* ≤ 0.05 relative to insulin-treated myotubes. *B*, C2C12 myotubes were incubated with or without 10 μmol/l C2-cer, 0.3 mmol/l oleate, or 0.3 mmol/l stearate for 2 h, or with or without 2 μmol/l A939572 for 16 h, before to be harvested. Fatty acids were assessed as described in the Experimental procedures section. Bars represent mean ± SEM (n = 6). ∗ represents significant change *p* ≤ 0.05 relative to the untreated control myotubes. *C*, C2C12 myotubes were incubated with 2 μmol/l A939572 for 16 h before to add 1 to 100 μmol/l C2-cer for 2 h. After 10 min insulin treatment (100 nmol/l), cells were lysed and lysates were immunoblotted with the indicated antibodies. Scanning densitometry was performed to quantify changes in ^473^Ser Akt, ^308^Thr Akt, and ^202^Thr/^204^Thr ERK abundance in cell lysates (n = 3). ∗ represents significant change *p* ≤ 0.05 relative to A939572-untreated myotubes. C2-cer, C2-ceramide.

To address this question, we pretreated C2C12 myotubes with 0.3 mM oleate 15 min before to add C2-cer. Two hours later, cells were incubated with insulin for 10 min and lysed. As previously shown, C2-cer completely prevented insulin to induce Akt phosphorylation (Fig. 6*A*). Interestingly, oleate prevented C2-cer action (Fig. 6*A*). In parallel, we followed another insulin target, the mitogen-activated protein kinase ERK, kinase whose activity was also downregulated in response to ceramide in muscle cells (22, 27). Figure 6*A* shows that, as observed with Akt, oleate prevented the inhibition of insulin-induced ERK phosphorylation by C2-cer. To reinforce the importance of oleate as a negative regulator of C2-cer action in myotubes, we artificially decreased intracellular oleate levels in the cells. For this purpose, we inhibited the stearoyl CoA desaturase 1 (SCD1), a critical enzyme involved in FA synthesis that introduces a cis-double bond in the Δ-9 position in its substrates, thereby converting stearate (18:0) to oleate (18:1). Figure 6*B* shows that C2-cer did not modify the unsaturated *versus* saturated free fatty acid (FFA) balance in the cells. In contrast, a SCD1 inhibitor (A939572) decreased this ratio, indicating an imbalance of unsaturated FFA towards saturated FFA. In addition, while oleate addition to the cells increased the intracellular oleate/stearate ratio, incubation of cells with the SCD1 inhibitor also decreased the oleate/stearate ratio by two-fold, a level observed when cells were incubated with 0.3 mM stearate alone (Fig. 6*B*). Of note, the inhibitor itself does not induce any significant cell cytotoxicity (+0.76% increase in cell death in cells treated with the inhibitor for 16 h compared to untreated cells (treated with DMSO alone, data not shown)). Interestingly, incubation of myotubes with the SCD1 inhibitor potentiated the negative action of C2-cer on both insulin-induced Akt and ERK phosphorylation (Fig. 6*C*) in a dose-dependent manner (Fig. S4*A*). It is important to note that preincubation of cells with the SFA stearate (C18:0) did not affect the action of C2-cer on insulin signaling (data not shown).

**Figure 7:**
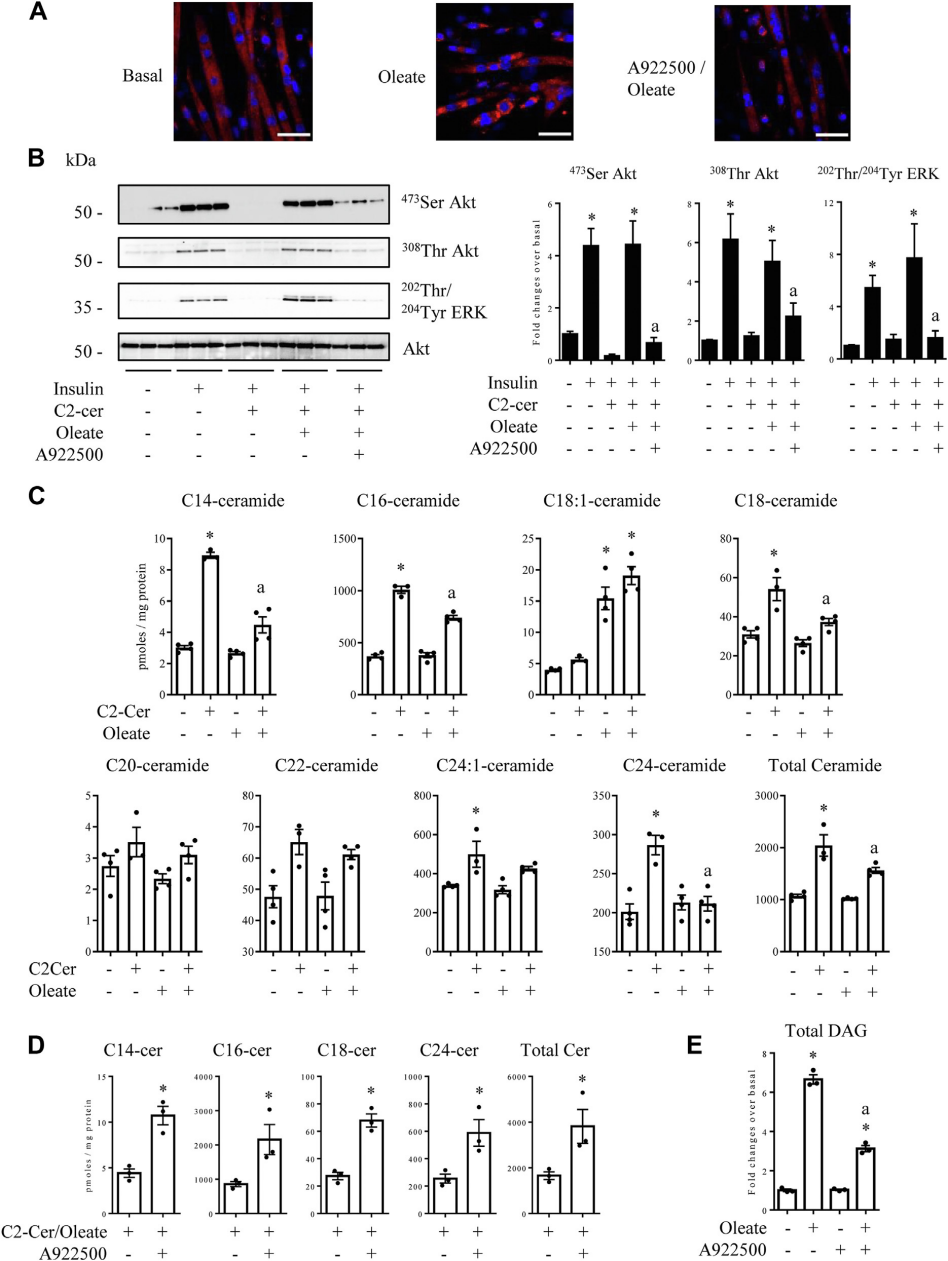
**Oleate channels fatty acids towards storage and prevents C2-cer to act on insulin signaling in C2C12 myotubes.***A*, C2C12 myotubes were incubated with 0.3 mmol/l oleate, in the presence or not in the presence of 10 μmol/l A922500 for 16 h before to fix the cells and to label TG (*red*) and nuclei (*blue*). Scale bar represents 50 μm. *B*, C2C12 myotubes were incubated with 10 μmol/l A922500 15 min before to add 0.3 mmol/l oleate. Thirty minutes later, cells were incubated for 2 h with 100 μmol/l C2-cer. After 10 min insulin treatment (100 nmol/l), muscle cells were harvested and lysates were immunoblotted with the indicated antibodies. Scanning densitometry was performed to quantify changes in ^473^Ser Akt, ^308^Thr Akt, and ^202^Thr/^204^Thr ERK abundance in cell lysates. Bars represent mean ± SEM (n = 3). ∗ represents significant change *p* ≤ 0.05 relative to the untreated control myotubes. ^a^ represents significant change *p* ≤ 0.05 relative to oleate/C2-cer/insulin–treated myotubes. *C*, C2C12 myotubes were incubated with 0.3 mmol/l oleate. Thirty minutes later, cells were incubated for 2 h with 100 μmol/l C2-cer. Ceramide content was assessed as described in the Experimental procedures section. Results are mean ± SEM (n = 3–4). ∗ represents significant change *p* ≤ 0.05 relative to untreated control myotubes. ^a^ represents significant change *p* ≤ 0.05 relative to C2-cer–treated myotubes. *D*, C2C12 myotubes were incubated with 10 μmol/l A922500 15 min before to add 0.3 mmol/l oleate. Thirty minutes later, cells were incubated for 2 h with 100 μmol/l C2-cer. Ceramide content was assessed as described in the Experimental procedures section. Results are mean ± SEM (n = 3). ∗ represents significant change *p* ≤ 0.05 relative to C2-cer/oleate–treated myotubes. *E*, C2C12 myotubes were incubated or not incubated with 10 μmol/l A922500 15 min before to add 0.3 mmol/l oleate for 2 h. DAG content was assessed as described in the Experimental procedures section. Results are mean ± SEM (n = 3). ∗ represents significant change *p* ≤ 0.05 relative to untreated control myotubes. ^a^ represents significant change *p*≤ 0.05 relative to oleate-treated myotubes. C2-cer, C2-ceramide; DAG, diacylglycerol.

Then, we looked for the mechanism by which oleate could prevent C2-cer to inhibit insulin signaling. C2C12 muscle cells presented a robust triglyceride synthesis in response to 16 h oleate incubation (Figs. 7*A* and S5). To test whether oleate may prevent C2-cer to be recycled into endogenous ceramide species by channeling lipogenesis-synthetized FAs into TAG, we inhibited diacylglycerol transferase 1 (DGAT1), one of the enzyme that catalyzes the final step in mammalian TG synthesis (46). In muscle cells, DGAT1 was shown to be dominant over DGAT2 to use FAs derived from *de novo*-synthesized FAs to generate TAG (46). In the presence of a DGAT1 inhibitor (A922500), cells accumulated much less lipids in response to oleate (Figs. 7*A* and S5). To determine whether the inability to accumulate TG could affect the inhibitory action of oleate on C2-cer action, we pretreated C2C12 muscle cells with the DGAT1 inhibitor, 15 min before to add oleate and then C2-cer for 2 h. Figure 7*B* shows that, as observed above (Fig. 6), oleate prevented C2-cer to inhibit insulin-induced both Akt and ERK phosphorylation. Pretreatment of cells with the DGAT1 inhibitor counteracted the preventive effect of oleate in a dose-dependent manner (Figs. 7*B* and S4*B*), suggesting that the ability to synthesize TG plays a critical role in protection from lipotoxicity. Like the SCD1 inhibitor, the DGAT1 inhibitor is not cytotoxic to cells (0.91% increase in cytotoxicity between myotubes treated with the inhibitor compared to those treated with DMSO only, data not shown). In parallel, and in the same conditions, we assessed C2-cer–induced endogenous ceramide content. Figure 7*C* shows that oleate prevented several ceramide species (C14-, C16-, C18-, and C24-cer) to be synthetized from C2-cer. It is interesting to note that basal C18:1-ceramide synthesis was sharply increased in response to oleate (Fig. 7*C*), suggesting that this ceramide species does not play by itself a critical role in regulating negatively insulin signaling.

To find out whether oleate-induced TAG synthesis is the mechanism by which C2-cer–induced endogenous ceramide content was prevented, we pretreated muscle cells with the DGAT1 inhibitor before to add both oleate and C2-cer. Then, we assessed cell endogenous C14-, C16-, C18-, and C24-cer content. Figure 7*D* shows that inhibition of TG synthesis indeed prevented oleate to reduce C2-cer–induced endogenous ceramide synthesis.

In parallel, oleate induced a 6-fold increase in DAG concentrations in muscle cells (Fig. 7*E*), and this increase was largely compromised in the presence of the DGAT1 inhibitor (Fig. 7*E*). These data therefore suggest also a lack of deleterious action of DAG on the insulin response in this muscle cell model.

## Discussion

Elevated levels of circulating SFAs observed during obesity promote their deposition into peripheral nonadipose tissues where they are rapidly transformed into various lipid species such as ceramides. This aberrant lipid production is now well known to mediate pathologies associated with obesity, T2D, and metabolic syndrome (3, 8, 16).

We have conducted this study on ceramide metabolism in C2C12 muscle cells. Although C2C12 myotubes are a mouse-derived cell line, they have been recognized as an effective research tool in numerous scientific aspects including aging, diabetes, obesity, hyperlipidemia, and muscle growth (47). C2C12 cells are easy to culture and they display several characteristics of human skeletal muscle cells such as glucose metabolism and insulin signaling machinery (17, 47). Thus, this cell line is convenient to assess the effect of lipotoxicity on muscle insulin response.

C2-cer was often used to study ceramide-mediated cellular process and particularly the effects of ceramide on insulin sensitivity. However, studies showed that SFAs such as palmitate did not produce only one type of ceramide but rather many different species, depending of the length of the FA added to the sphingoid backbone by different CerS (48). The generation of endogenous ceramides from C2-cer was already described in human endothelial cells where a 2-fold increase in both sphingosine and long-chain ceramide was observed after a 4 h incubation of the cells with C2-cer (49). We show here in C2C12 myotubes that generation of endogenous long-chain ceramides from C2-cer involves a process of deacylation/re-acylation, respectively, by ceramidase and CerS activities, that use FAs produced from endogenous lipogenesis. Importantly, these newly formed ceramides are responsible for the loss of cell insulin response. In addition, a monounsaturated FA such as oleate, already described to counteract palmitate-induced ceramide *de novo* synthesis (42, 43, 44), can also prevent C2-cer to be recycled into endogenous ceramide species in muscle cells.

Interestingly, and in opposite to what we observe in response to C2-cer incubation, accumulation of endogenous ceramide after treatment of myotubes with the ceramidase inhibitor ceranib-2 alone does not lead to an inhibition of insulin signaling. These data suggest that endogenous ceramide accumulated after ceramidase inhibition or produced from C2-cer are not equivalent to induce insulin resistance, which can be potentially related to a different intracellular localization. This observation is not unprecedented as it has already been shown in MCF7 breast cancer cells that a selective accumulation of ceramides in mitochondria induced cell death, while their targeting to other intracellular compartment such as plasma membrane, Golgi apparatus, ER, or nucleus was not effective (50). In addition, exposure of human glioma cells to ceranib-2 was more efficient to induce apoptosis than C2-cer, demonstrating that ceramides produced by ceranib-2 were more cytotoxic than ceramides derived from C2-cer (51). Interestingly, a recent study showed that only sarcolemmal ceramides were strongly related to insulin resistance and hyperinsulinemia/hypertriglyceridemia (52). In the mitochondrial/ER and nuclear fractions, however, ceramides were not correlated with insulin sensitivity (52). This and our data suggest that a precise pool of ceramide subcellular localization is crucial for a negative action on insulin signaling.

In our muscle cell model, we find that CerS are mainly using FA produced by *de novo* lipogenesis to recycle sphingosine generated from C2-cer catabolism into long-chain ceramide species. It must be emphasized, however, that in cultured cells, lipogenesis from glucose is usually the only way to provide FAs. Thus, its importance, when compared to the *in vivo* situation, might then be increased. Nevertheless, these data suggest that, *in vivo*, muscle lipogenesis could play an important role in ceramide production and, subsequently, in the regulation of muscle insulin sensitivity. Interestingly, mice deficient for muscle *de novo* lipogenesis (FAS KO mice) are protected from muscle insulin resistance induced by a high fat diet (53). The *in vivo* role of muscle lipogenesis on ceramide biosynthesis must be further explored in this context.

Another interesting finding is to show that the monounsaturated FA oleate can counteract the recycling of C2-cer into endogenous ceramide species in muscle cells. Previous studies showed that oleate was able to shift palmitate metabolism towards FA β-oxidation and TAG production at the expense of both DAG and ceramide synthesis (42, 43, 44) through an increase in carnitine palmitoyltransferase 1 (44) and/or DGAT expression (43) in muscle cells. In addition, oleate downregulates the expression of several enzymes involved in ceramide biosynthesis (11, 45). However, short-term treatment with oleate (2 h) suggests that the inhibitory mechanism does not involve a transcriptional mechanism. The inhibitory action of oleate is not due to a shift toward the synthesis of C18:1-ceramide since this FA is a weak substrate for CerS. We show here that oleate stops C2-cer recycling by channeling FA towards esterification into TG. Indeed, inhibition of DGAT1, which prevents TG synthesis, was sufficient to amplify C2-cer recycling and insulin resistance in muscle cells. This result is in agreement with a pioneering study which showed that high levels of oleate could drastically increase DGAT1 activity in Sf9 insect cells overexpressing DGAT1 (54).

Importantly, we find that decreasing the ratio of oleate/stearate with an SCD1 inhibitor in muscle cells favors the recycling of C2-cer and potentiated the inhibitory action of C2-cer on insulin signaling. Overall, our data suggest that monounsaturated FAs such as oleate act at different levels to prevent ceramide accumulation in muscle cells. Oleate improves mitochondrial β-oxidation of SFAs and also inhibits exogenous ceramide to be recycled into other harmful ceramide species. How regulation of TG synthesis by oleate does inhibit ceramide recycling? Like CerS isoforms, DGAT1 is an ER membrane protein (55) and it has been recently shown that DGAT1 protects ER from lipotoxicity and adipose tissue inflammation through TG synthesis (56). Therefore, a competition for FA between CerS and DGAT1 could exist, and by promoting FA esterification into TG, oleate could block sphingosine acylation.

Compared to the *de novo* sphingolipid synthesis pathway, regulation of the salvage pathway is less studied, even if the implication of this pathway as a supplier of ceramide molecules during lipotoxicity has already been suggested. Indeed, some negative effects of palmitate on pancreatic β-cells have been attributed to the generation of ceramide from sphingosine acylation (11). In addition, inhibition of sphingomyelin hydrolysis protects skeletal muscle cells from SFA-induced insulin resistance and inflammation (10). Overall, our present results suggest a role in the salvage recycling of complex sphingolipids into ceramide as potential contributor to the induction of insulin resistance.

In view of our data, an important issue to resolve remains the identity of the ceramide species responsible for the loss of insulin response observed in response to C2-cer. Our data show that concentrations of C14:0, C16:0, C18:1, C18:0, C24:0, and C24:1-ceramide species are increased in response to C2-cer recycling in myotubes (Fig. 1*D*). While C18:1-cer is clearly not a candidate (see Fig. 7*C*), the other species (and mainly C16- and/or C18:0-cer) are known insulin resistant lipids (57). A study by Obeid's group showed in human breast adenocarcinoma cells that CerS5/6 played an important role in ceramide synthesis (mainly C14/16-cer) using the sphingosine salvage pathway (58). Is this also the case in our myotube model? Future work will be needed to answer this question.

It has been shown that increased content of circulating low density lipoproteins-ceramides secreted by the liver was observed in T2D patients compared to healthy individuals and promotes insulin resistance (59). Increased plasma ceramide/dihydroceramide levels have been described as predictive biomarkers of T2D appearance (60). Therefore, it is tempting to speculate that, in addition to ceramides produced *de novo*, endocytosis and recycling of exogenous circulating low density lipoproteins-derived ceramides could simultaneously affect the insulin response and therefore glucose homeostasis in muscles.

A final point of interest in our study concerns the changes in DAG concentration in myotubes in response to oleate. Although oleate induces a significant increase in intracellular concentrations of total DAG, no negative impact was observed on insulin response. This result is at variance with studies showing a negative influence of DAG on insulin action (61, 62). One explanation could be the short time of treatment with oleate (2 h), not long enough to allow the synthesis of the right detrimental DAG species. Another possibility could be directly related to the type of DAG species produced in response to oleate. Indeed, under our conditions, more than 99% of the DAGs produced are 18:1/18:1-DAG (coming specifically from oleate, data not shown). In contrast with ceramides, the identity of the DAG species responsible for muscle insulin resistance is not firmly established. However, one study showed that both 18:0/18:2-DAG and 18:2/18:2-DAG could be associated with muscle insulin resistance (63). In our model, concentrations of these two DAG species did not change in response to oleate (data not shown). This result could explain why DAGs produced in response to oleate are not deleterious under our conditions.

The second observation concerns the effect of the DGAT1 inhibitor on intracellular DAG concentrations in the presence of oleate. Since DGAT1 catalyzes the final step of TG synthesis, the conversion of DAG to TG, a decrease in intracellular DAG concentrations was unexpected in response to the DGAT1 inhibitor. However, such an outcome was already observed in other studies. In the heart, DGAT1 inhibition did not induce an increase in DAG concentrations but rather a decrease (64). And another study showed that the specific injection of DGAT1 gene construct in rat *tibialis anterior* muscle induced an increase in muscle DAG amounts (65). These observations, as well as ours, go against what was observed in transgenic mice overexpressing DGAT1 where an increase in intramuscular lipid concentrations was accompanied with a decrease in muscle DAG concentrations (66). Two possibilities exist to explain these differences. Our study was performed *in vitro* and, like the one overexpressing DGAT1 specifically in *tibialis anterior* muscle of rats, is truly muscle-specific. Secondly, when TG synthesis is inhibited, it could preferentially channel DAG towards other lipids species such as phosphatidylcholine, phosphatidylethanolamine, and sphingomyelin (67). This may be a way to prevent DAG accumulation in cells. More study is needed to understand this regulation.

In summary, this study validates C2-cer as a convenient tool to decipher mechanisms by which SFA mediate insulin resistance in muscle cells since it increases endogenous ceramide levels responsible of insulin resistance. In addition, it provides important mechanistic data showing for the first time that C2-cer induces a loss in insulin sensitivity in muscle cells through its recycling into endogenous ceramide species *via* a salvage-recycling pathway. This later recycling model opens a novel potential role of the uptake/recycling of circulating ceramide in the onset of insulin resistance.

## Experimental procedures

### Materials

All reagent-grade chemicals including insulin, TOFA, FB1, myriocin, ceranib-2, A939572, oleate, and stearate were obtained from Sigma-Aldrich. C2-cer was obtained from Merck Chemicals. C-75 was from Tocris Bioscience. Antibodies against ^473^Ser-Akt, ^308^Thr-Akt, native PKB/Akt, GLUT4, and α1 NA-K-ATPase were from Cell Signaling Technology, and antibody against ceramide (MID 15B4 clone) was from Enzo Life Sciences. Horseradish peroxidase anti-rabbit and horseradish peroxidase anti-mouse were from Jackson ImmunoResearch Laboratories, and the enhancer chemiluminescent Supersignal was from Thermo Fisher Scientific. Ceramide secondary antibody (Alexa fluor 488 goat anti-mouse) was from Thermo Fisher Scientific. Glucose uptake fluorometric assay kit was from Sigma-Aldrich.

### Cell culture

C2C12 myoblasts were grown in Dulbecco’s modified Eagle’s medium (Gibco), containing 10% fetal bovine serum (Gibco) and 1% penicillin/streptomycin (Gibco) at 37 °C in a humid atmosphere with 10% CO2. After being grown to confluence, they were differentiated into myotubes by replacing the medium with Dulbecco’s modified Eagle’s medium containing 2% horse serum (Gibco) and 1% penicillin/streptomycin. Cells were treated with palmitate conjugated to FA-free bovine serum albumin (BSA) as previously described (68).

### Preparation of whole cell lysates

C2C12 myotubes were lysed as described previously (69) following experimental manipulation (see figure legends), in an appropriate volume of lysis buffer (50 mM Tris, pH 7.4, 0.27 M sucrose, 1 mM Na–orthovanadate, pH 10, 1 mM EDTA, 1 mM EGTA, 10 mM Na-glycerophosphate, 50 mM NaF, 5 mM Na-pyrophosphate, 1% (w/v) Triton X-100, 0.1% (v/v) 2-mercaptoethanol, and protease inhibitors). Whole cell lysates were centrifuged (10,000*g*, 4 °C for 10 min) and stored at −80 °C until required.

### Immunoblotting

Proteins from cell lysates were separated by SDS-PAGE on 10% or 14% polyacrylamide gels and were transferred to nitrocellulose membranes (Thermo Fisher Scientific). Nitrocellulose membranes were probed with various antibodies. Detection of primary antibodies was performed using appropriate peroxidase-conjugated IgGs, and protein signals were visualized using enhanced chemiluminescence.

### Analysis of sphingolipid species content

Ceramide, sphingosine, and S1P contents were measured using an UHPLC/MS/MS approach. Briefly, samples were homogenized in a buffer composed of 0.25 mol/l sucrose, 25 mmol/l KCl, 50 mmol/l Tris, and 0.5 mmol/l EDTA, pH 7.4. Immediately afterward, internal standards (17C-sphingosine, 17C-S1P, d17:1/8:0, d17:1/18:0, d17:1/18:1(9Z), d17:1/20:0, d17:1/24:0, and d17:1/24:1(15Z) (Avanti Polar Lipids) as well as extraction mixture (isopropanol:water:ethyl acetate, 30:10:60; v:v:v) were added to each homogenate. The mixture was vortexed, sonicated, and then centrifuged. The supernatant was transferred to a new tube and pellet was re-extracted. After centrifugation, supernatants were combined and evaporated under nitrogen. Dried samples were reconstituted in LC solvent B (2 mmol/l ammonium formate, 0.15% formic acid in methanol) for UHPLC/MS/MS analysis. Sphingolipid content was analyzed by means of triple quadrupole mass spectrometer using positive ion electrospray ionization source (except for S1P, which was analyzed in negative mode) with multiple reaction monitoring against the concentration standard curves. In some experiments, ceramide concentration was measured by the DAG kinase enzymatic method as previously described (70). Briefly, lipid extracts were incubated in the presence of *Escherichia coli* DAG kinase and [γ-^32^P]-ATP. Reaction was stopped, and [γ-^32^P]-ceramide phosphate was resolved by TLC with chloroform/acetone/methanol/acetic acid/water (10:4:3:2:1, by vol.) and quantified using a FLA700 PhosphorImager (GE Healthcare). An aliquot was used to quantify total phospholipid levels as described previously.

### Analysis of DAG

Cell lysates were spiked with 1.36 μg of 1,3 diheptadecanoyl-glycerol (d5) (Avanti Polar Lipids) and used as internal standard. Folch extracts (4 μl) were injected in a 1260 LC system coupled to a 6460 triple quadrupole mass spectrometer operating in positive APCI mode (LC parameters: column Poroshell C8 2.1 × 100 mm, 2.7 μm; solvent A: methanol/acetonitrile/ammonium acetate, 10 mM 60/20/20; solvent B: MTBE/isopropanol/ammonium acetate, 10 mM 22/74/4; 0.4 ml/min; 50 °C; linear gradient from 5% B to 75% B in 16 min; source parameters: Gas and APCI heater temperatures 250 °C, gas flow 4 ml/min, nebulizer 50 psi, capillary voltage 4000 V, APCI voltage 4 V, MS parameters: fragmentor 148 V, collision energy 23 V). Analyses were performed in selected reaction monitoring mode from the [DAG+NH4]^+^ precursor ion to the [M-*sn2* FA]^+^ product ion. Relative quantitation of DAG was performed by calculating the response ratio of the considered DAG to (17:0-d5)_4_-DAG used as internal standard.

### Measurement of FFA levels by GC-MS

Briefly, and as described previously (71), cells were mixed with BF3 (14%)/methanol, and 10 mg of heptadecanoic acid as an internal standard were added. Samples were heated (100 °C; 40 min) and then cooled at room temperature. Heptane/distilled water (1:2) was added, and samples were centrifuged. The supernatant was collected and evaporated with a Speedvac (Jouan). Dry samples were solubilized in heptane, and 1 μl FA methyl esters was analyzed on GC2010 instrument. The mass spectra and retention indices registered in the FA methyl esters’ GC/MS library were obtained using the Shimadzu GCMS-QP2010.

### Immunofluorescence

C2C12 myotubes were incubated for 2 h with C2-cer (50 μM), washed twice with PBS, fixed with 4% paraformaldehyde for 15 min at room temperature, and blocked for 15 min with PBS, 1% BSA, 0.01% Triton X-100. Cells were incubated with the ceramide antibody overnight at 4 °C and the following day with secondary antibody for 1 h (Alexa Fluor 488 goat anti-mouse, Thermo Fisher Scientific) and DAPI. Labeling patterns were observed using the Zeiss LSM 710 confocal microscope. Images were processed using Image J software (https://imagej.net/software/imagej/).

### Subcellular fractionation

C2C12 plasma membrane fractions were isolated as described previously (69). Briefly, 1 h before fractionation, cells were scraped with a rubber policeman, pooled, and gently pelleted. The cell pellet was resuspended in ice-cold buffer (250 mmol/l sucrose, 20 mmol/l Hepes, 5 mmol/l NaN_3_, 2 mmol/l EGTA, 100 μmol/l PMSF, 10 μmol/l *trans*-epoxysuccinyl-L-leucyl amido [4-guanidino]butane, and protease inhibitors; pH 7.4) and homogenized. Then, the cellular homogenate was subjected to a series of differential centrifugation steps to isolate crude plasma membranes, which were subsequently fractionated on a discontinuous sucrose gradient (32, 40, and 50% sucrose by mass) at 210,000*g* for 2.5 h. Membranes recovered from the top of the 32% sucrose cushion were enriched in plasma membranes and isolated for further analyses. The protein content of the isolated membrane fractions was determined with the Bradford assay using BSA as the standard (72).

### Glucose uptake

We used the glucose uptake Fluorometric Assay Kit from Sigma-Aldrich following the manufacturer’s instructions. Briefly, glucose uptake in C2C12 myotubes was measured using a glucose analog, 2-DG. 2-DG uptake was determined by a coupled enzymatic assay in which the 2-DG6P is oxidized, generating NADPH which reacts with the probe to generate a fluorometric signal (λ_ex_535/λ_em_587 nm) proportional to the 2-DG taken up by the cells.

### Real time PCR

Total RNA was isolated from C2C12 cells using Trizol (Invitrogen) according to the manufacturer's instructions. The RNA concentration was measured with the Nanodrop 2000 spectrophotometer (Thermo Fisher Scientific). The isolated RNA (1 μg from each sample) was reverse-transcribed to complementary DNA (cDNA) by MMLV reverse transcriptase (Invitrogen) according to the manufacturer's instructions. Complementary DNAs were amplified using iTaq DNA Polymerase (Bio-Rad), and the primers are listed in Table 1. Conditions for the PCR were one cycle of 95 °C for 15 s, 39 cycles of 95 °C for 15 s, 60 °C for 30 s, and 55 °C for 30 s. The mRNA expression levels were normalized by using gene cyclophilin as the internal standard.

**Table 1:** Mouse primers used throughout the study

Gene	Forward primer (5′ to 3′)	Reverse primer (5′ to 3′)
Cyclophilin	TGGAGAGCACCAAGACAGACA	TGCCGGAGTCGACAATGAT
CerS1	TGACCCGCCCTCTGTCTTC	ACCACCGAGTCCTTACGCC
CerS2	CAGCTCTGCACCGGACG	GGTTAAGTTCACAGGCAGCCAT
CerS3	ACATATCTCCCTTTGCCCTGATG	ATAATTGCAAGAGACGGCAATGA
Cers4	CATGACTGCTCCGACTACCTG	GAATATGAGGCGCGTGTAGAA
CerS5	CTACCTAATTGTCCAGACTGCTTCC	AGAGAGGTTGTTTTTGTGGGTTG
CerS6	TTGCAAAACTGTTTCAAAAGGC	ACCGCTGGTTCCGTTGGT
Acer1	TGATGCTTGACAAGGCACCA	GGCAATTTTTCATCCACCACC
Acer2	AGTGTCCTGTCTGCGGTTACG	TGTTGTTGATGGCAGGCTTGAC
Acer3	GATTCACTGAGGAACTTTCG	AGAGAAACTTCACTTTTGGC

### Neutral lipid staining

C2C12 myotubes were washed with PBS and fixed with 4% paraformaldehyde for 15 min. LipidTOX neutral lipid stain (Thermo Fisher Scientific) and DAPI were added. Cells are incubated 30 min at room temperature before imaging. Labeling patterns were observed using the Zeiss LSM 710 confocal microscope.

### Lipid extraction and triglyceride assay

C2C12 cells were washed with PBS, homogenized in a chloroform:methanol mixture (2:1), and incubated at 37 °C overnight with gentle shaking. Cells were centrifuged for 10 min at 1500*g*, and the supernatant is resuspended in NaCl 0.9%. After a vigorous vortexing and a centrifugation, the bottom layer is transferred into a new glass tube and evaporated under a nitrogen flow. The pellet is resuspended in 200 μl of isopropanol. The triglycerides assay was performed with the TRIGS Assay (RANDOX). The standard range is from 0 to 40 μg/μl by diluting the calibrator. Two microliters of the standard or the sample was added into a 96-wells plate. Two hundred microliters of the enzyme mixture was added. The plate is incubated at 37 °C for 10 min, and the absorbance is measured at 500 nm using the SPARK 10 M spectrophotometer (TECAN).

### Cytotoxicity assay

The cytotoxic effect of A939572 was measured by 3-[4,5-dimethylthiazol-2-yl]-2,5 diphenyl tetrazolium bromide (MTT) assay. The MTT assay is based on the conversion of MTT into formazan crystals by living cells, which reflects mitochondrial activity. C2C12 cells were seeded at a concentration of 2 × 10^3^ cells/well in 100 μl culture medium containing various concentrations of A939572 in a 96-wells plate at 37 °C, 5% CO_2_. Ten microliters of the MTT labeling reagent (final concentration 0.5 mg/ml) (Merck) was added to each well. After 4 h of incubation at 37 °C, 100 μl of solubilization buffer were added. The absorbance is measured at 570 nm with a reference wavelength at 630 nm using the SPARK 10 M spectrophotometer (TECAN).

### Statistical analysis

Data were analyzed with GraphPad Prism 6.07 statistical software using unpaired two-tailed *t* test (two groups) or one-way ANOVA. Data were considered statistically significant at *p* values ≤ 0.05.

## Data availability

All data are contained in the article.

## Supporting information

This article contains supporting information.

## Conflict of interest

The authors declare that they have no conflicts of interest with the contents of this article.
