# Supplementary material for: Ceramide analog C2-cer induces a loss in insulin sensitivity in muscle cells through the salvage/recycling pathway
Source: J Biol Chem. 2023 May 11;299(6):104815. doi: 10.1016/j.jbc.2023.104815 (PMC10276168; doi:10.1016/j.jbc.2023.104815)
Supplement: Supporting Figures S1–S5 [file mmc1.pdf]

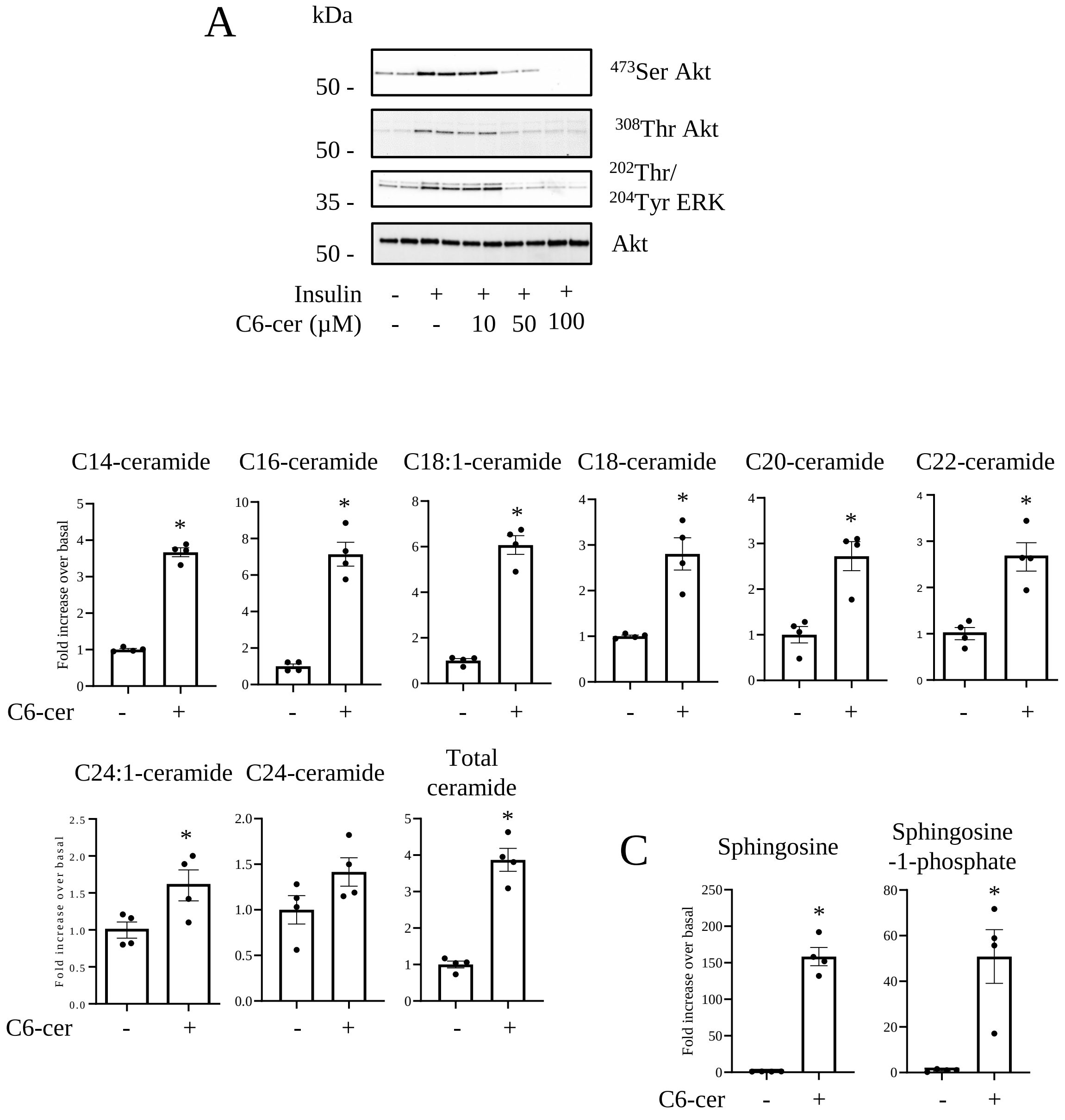

Supporting figure 1: Effect of C6-ceramide on insulin response and endogenous ceramide synthesis in C2C12 myotubes.

(A) C2C12 myotubes were incubated with 10 to 100 µmol/l C6-ceramide (C6-cer) for 2 h followed by 100 nmol/l insulin for 10 min before cell lysis. Cell lysates were immunobloted with the indicated antibodies. (B) C2C12 myotubes were incubated with 100 µmol/l C6-cer for 2h. Following these incubations, muscle cells were harvested to assess ceramide content as described in the Methods section. Results are mean +/- SEM (n=4). * Significant change p≤ 0.05 relative to the untreated control myotubes.

A

B

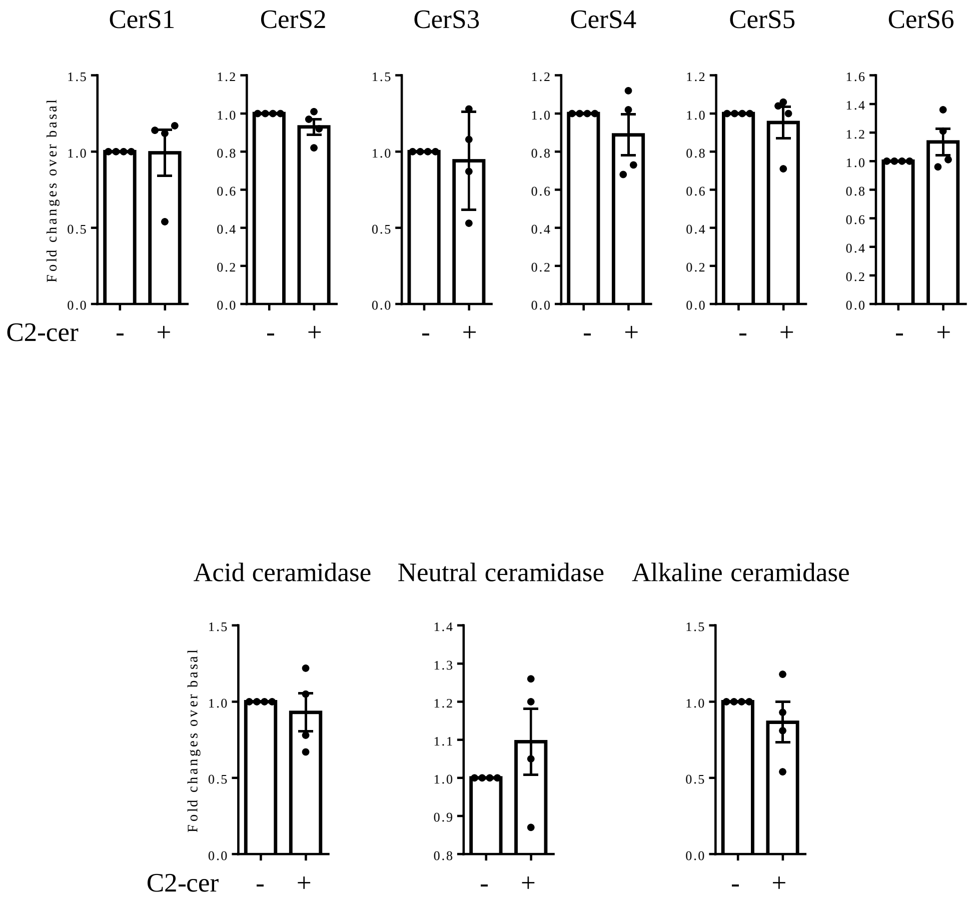

Supporting figure 2: Effect of C2-cer on the expression of CerS and ceramidase isoforms.

C2C12 myotubes were incubated for 2h with 100 µmol/l C2-ceramide (C2-cer). (A) Quantification of CerS1, CerS2, CerS3, CerS4, CerS5 and CerS6 levels was performed by qRT-PCR. (B) Quantification of acid, neutral and alkaline ceramidase levels was performed by qRT-PCR. Results are means +/- SEM (n=4).

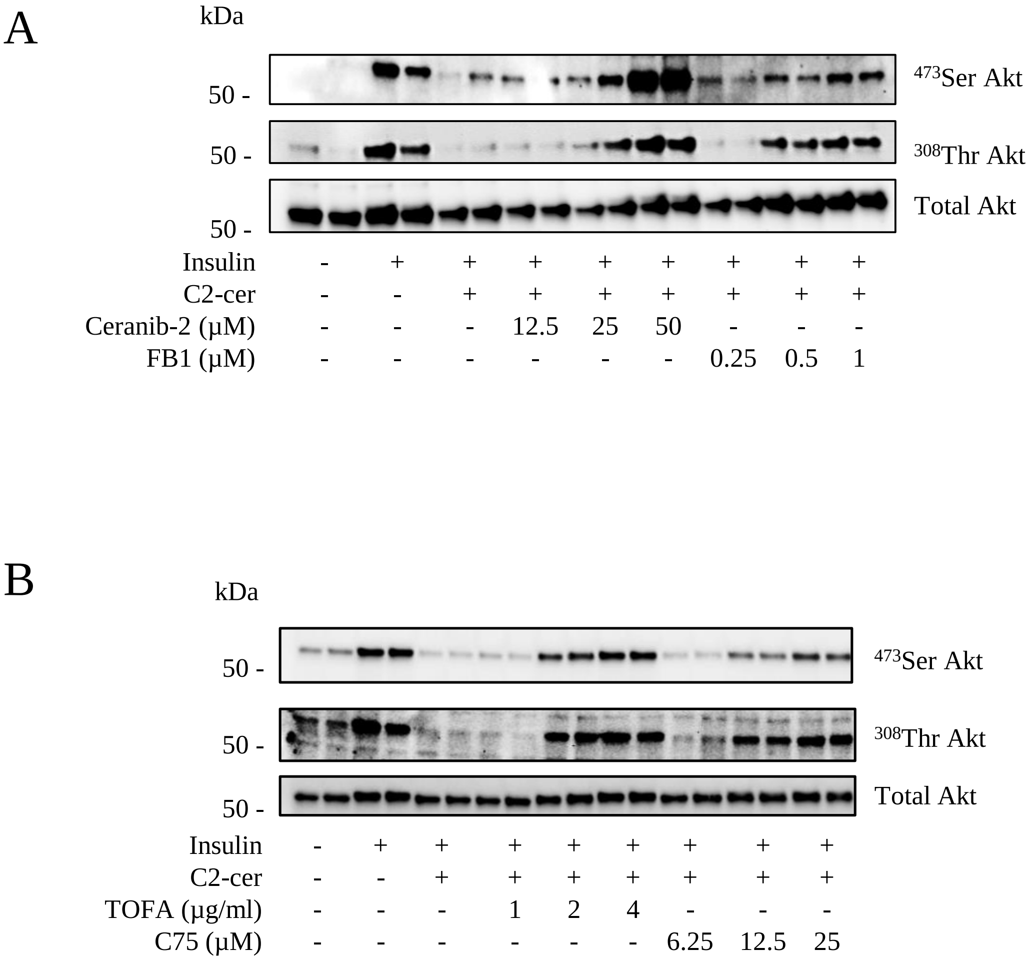

Supporting figure 3: Dose-response effects of ceranib-2, FB1, TOFA or C75 on the negative action of C2-cer on insulin response in C2C12 myotubes.

C2C12 myotubes were incubated with 100 µmol/l C2-cer for 2 h in the presence of (A) ceranib-2 (12.5 to 50 µM) or FB1 (0.25 to 1 µM), (B) TOFA (1 to 4 µg/ml) or C75 (6.25 to 25 µM), followed by 100 nmol/l insulin for 10 min before being lysed. Cell lysates were immunoblotted with the indicated antibodies.

A

B

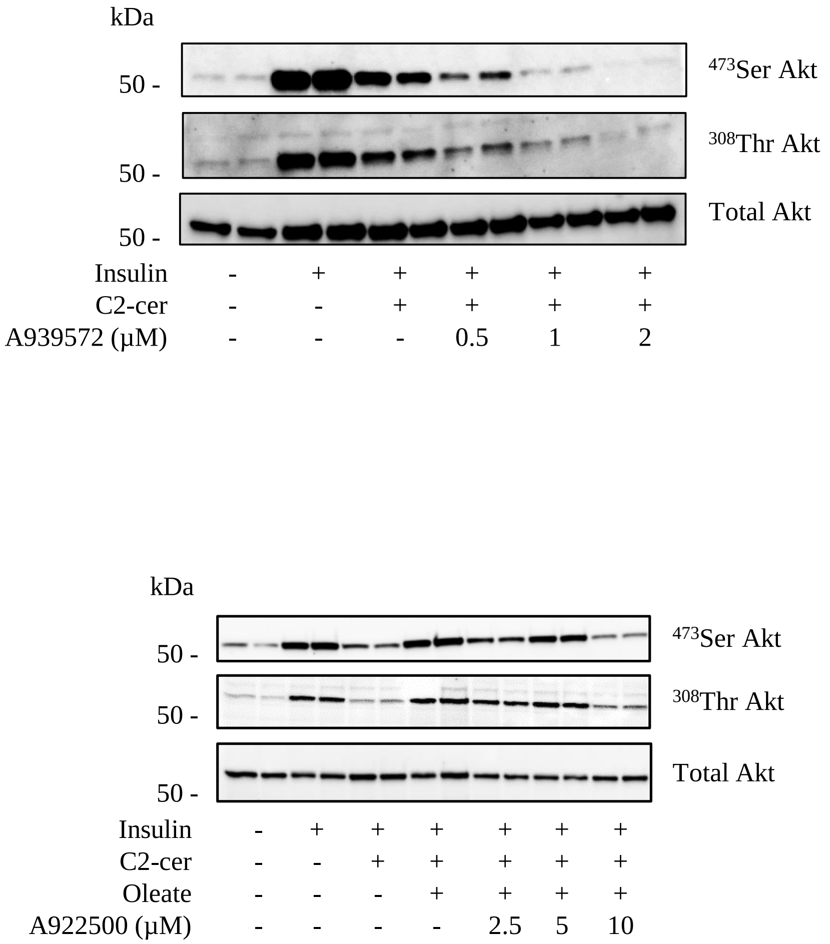

Supporting figure 4: Dose-response effects of A922500 or A939572 on the negative action of C2-cer on the insulin response in the presence of oleate in C2C12 myotubes. (A) C2C12 myotubes were incubated with A939572 (0.5 to 2 µM) for 16 h before to add 50 µmol/l C2-cer for 2h. (B) C2C12 myotubes were incubated with A922500 (2.5 to 10 µM) 15 min before to add 0.3 mmol/l oleate. 30 min later, cells were incubated for 2h with 100 µmol/l C2-cer. After 10 min insulin treatment (100 nmol/l), muscle cells were harvested and lysates were immunoblotted with the indicated antibodies.

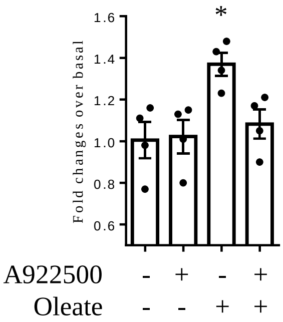

Supporting figure 5: Effect of oleate on TG synthesis in C2C12 myotubes.

(A) C2C12 myotubes were incubated with 0.3 mmol/l oleate in the presence or not of 10 µmol/l A922500 for 2 h before cells were harvested and TG assessed as described in the Methods section. Results are mean +/- SEM (n=4). * Significant change p≤ 0.05 relative to the untreated control myotubes.
